# Comparison of anesthetic efficacy of 4% articaine with 1:100,000 epinephrine and 2% lidocaine with 1:80,000 epinephrine for inferior alveolar nerve block in patients with irreversible pulpitis

**DOI:** 10.4317/jced.51617

**Published:** 2014-12-01

**Authors:** Ravi Sood, Manoj-Kumar Hans, Shashit Shetty

**Affiliations:** 1 MDS, (Conservative Dentistry and Endodontics), Private Practitioner. New Delhi, India; 2 MDS, Associate Professor. Department of Conservative Dentistry and Endodontics, Vyas Dental College and Hospital, Jodhpur, Rajasthan, India; 3 MDS, Professor and Head of Department. Department of Conservative Dentistry and Endodontics, K.D.Dental College, Mathura, U.P, India

## Abstract

Objectives: This study was done to compare the anesthetic efficacy of 4% articaine with 1:100,000 epinephrine with that of 2% lidocaine with 1:80,000 epinephrine during pulpectomy in patients with irreversible pulpitis for inferior alveolar nerve block in mandibular posterior teeth. Material and Methods: Patients with irreversible pulpitis referred to the Department of Conservative Dentistry and Endodontics, K.D. Dental College, randomly received a conventional inferior alveolar nerve block containing 1.8 mL of either 4% articaine with 1:100,000 epinephrine or 2% lidocaine with 1:80,000 epinephrine. After the patient’s subjective assessment of lip anesthesia, the absence/presence of pulpal anesthesia through electric pulp stimulation was recorded and the absence/presence of pain was recorded through visual analogue scale. Results: The pulpal anesthesia success for articaine (76%) was slightly more than with lidocaine (58%) as measured with pulp tester as well as for the pain reported during the procedure the success rate of articaine (88%) was slightly more than that of lidocaine (82%) although the difference between the two solutions was not statistically significant. Conclusions: Both the local anesthetic solutions had similar effects on patients with irreversible pulpitis when used for inferior alveolar nerve block.

** Key words:**Anesthesia, articaine, lignocaine, pulpitis.

## Introduction

Articaine comparatively is a new anesthetic, which has recently been introduced in Brazil and was approved for use in the United States in April 2000 (1). It is the most commonly used local anesthetic in Canada and in several European countries (2,3). In Germany, it accounts for 80% of all local anesthetics used for endodontic treatments (4,5).

Articaine is the center of heated discussions among dental surgeons as it has a faster onset and higher success rates than lidocaine as well as it has been demonstrated to increase the risk of paresthesia which could not be confirmed in a recent study (6-8). Articaine has been shown to be a significantly better anesthetic agent as compared to lidocaine for infiltrations (9-11).

 So far, research on the anesthetic efficacy of articaine in patients with irreversible pulpitis for inferior alveolar nerve block has been very limited (12-14). Therefore, to contribute to a more profound knowledge about the quality of articaine as a local anesthetic for endodontic treatments, the purpose of the present study was to compare the anesthetic efficacy of 4% articaine with 1:100,000 epinephrine with 2% lidocaine with 1:80,000 epinephrine for IAN blocks in patients with mandibular posterior teeth experiencing pulpitis.

## Material and Methods

One hundred adult patients [n=100] aged between 18-50 years participated in this prospective, randomized, double-blind clinical study. The patients included in this study had been admitted to the Department of Conservative Dentistry and Endodontics, K.D. Dental College with a clinical diagnosis of irreversible pulpitis. The patients had at least 1 adjacent tooth plus a healthy contralateral canine or, alternatively, a contralateral canine without deep carious lesions, extensive restoration, advanced periodontal disease, history of trauma, or sensitivity and gave a positive response on electric pulp testing of the diseased tooth and a prolonged response with moderate to severe pain to a cold testing using Roeko Endo-Frost [Roeko, Langenau, Germany]. While Patients who took medication potentially interacting with any of the anesthetics or with systemic disorders, history of sensitivity to anesthetic agents, presence of periodontal ligament [PDL] widening or periapical radiolucency or pregnancy were not included.

The study was approved by the Committee on the Ethics of Research on Human Beings and each patient gave written informed consent to participate in the study. Before the IAN block injections, the tooth with irreversible pulpitis, the adjacent tooth, and the contralateral canine were tested for pulp vitality with an electric pulp stimulator. The electric pulp stimulation of the contralateral canine, which had not been anesthetized, was used as control to ensure that the equipment was working properly and that patients were responding adequately.

The 100 participants were divided into 2 groups of 50 patients, who received IAN injections of 1.8 mL [equivalent to 1 cartridge] of either 2% lidocaine with 1:80,000 epinephrine or 4% Articaine with 1:100,000 epinephrine.

To ensure the blindness of the study, the label on the cartridges was removed and the cartridges were coded. 1 cartridge [1.8 mL] of either anesthetic solution was sealed in envelopes. At the time of application, one researcher, who administered the anesthesia injections, chose one of the envelopes at random. Electric pulp stimulations to assess pulpal anesthesia were performed by a colleague to guarantee that the anesthetic solution remained unknown and thus maintain the double-blindness of the study. Before the IAN block injections, the tooth with irreversible pulpitis, the adjacent tooth, and the contralateral canine were tested for pulp vitality with an electric pulp stimulator.

For the injections, a side-loading carpule syringe fitted with a 27-gauge 0.4 X 35 mm needle and equipped with a blood aspiration device and a thumb ring was used. Blood aspiration tests were carried out before each anesthesia injection as well as when changing needle position.

The cartridge of the respective anesthetic solution was applied as follows:

The index finger and thumb were used to palpate over the anterior border of the ramus of the mandible until the coronoid notch is felt. The palpating finger was then moved lingually across the retromolar triangle and onto the internal oblique ridge. The syringe was directed from the premolar region of the opposite side, and the needle was inserted until establishing bone contact. Thereafter, the needle was withdrawn 1–2 mm, the blood was aspirated, and the remaining 1.5 ml of anesthetic solution was slowly injected. The needle was then withdrawn to half its length and brought on the same side and remaining 0.3 ml was injected to anesthetise the lingual nerve. The average injection time for each cartridge was approximately 2 minutes.

Ten minutes after the IAN block, subjective lip anesthesia was evaluated by asking the patient whether his/her lip was numb. Thereafter and immediately before the pulpectomy, the electric pulp stimulations were repeated to determine pulpal anesthesia. During the pulpectomy procedure, the patients were instructed to report any painful discomfort.

To evaluate the intensity of pain during the pulpectomy, a visual analogue scale was used: 0, no pain; 1, mild, bearable pain; 2, moderate, unbearable pain; 3, severe, intense, and unbearable pain. The anesthesia was defined as successful when the dentist accessed the pulp chamber without pain being reported by the patient [pain scores 0 or 1]. In these cases, the pulpectomy procedure was continued. Pain scores of 2 or 3 classified the IAN block as unsuccessful. In these cases, an intrapulpal anesthesia was performed, and the pulpectomy was finalized. This complementary anesthesia was not evaluated, because it was beyond the scope of this study.

The responses to the electric pulp tester [negative or positive] and the pain [“with pain,” scores 2 or 3, or “without pain,” scores 0 or 1] recorded in the 2 test groups [articaine solution and lidocaine solution groups] were compared by using the χ2 test. Potential differences in age between the 2 groups were analyzed with T-test. The Mann-Whitney U test was used to compare the distributions of types of teeth with irreversible pulpitis in both groups. For all performed tests, the level for significance of differences was taken as *P* < .05.

## Results

In the present study, there were no statistically significant differences between the patients of the 2 test groups [articaine versus lidocaine solution] concerning gender distribution [articaine group, 60% female; lidocaine group, 46% female; *P* = 0.161], age [average age: articaine group, 26.46 years; lidocaine group, 28.90 years; *P*=0.233], and the types of teeth with irreversible pulpitis [*P* = 0.188] ( Table 1).

**Table 1:**
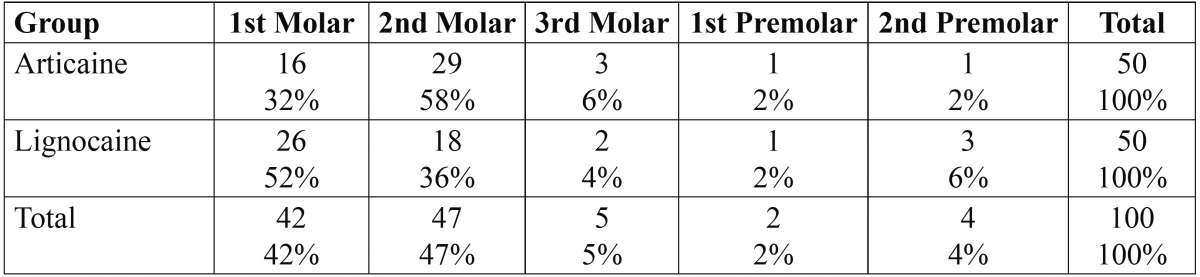
Types of Teeth with Irreversible Pulpitis.

Mann-Whitney U test was also carried out for the types of teeth with irreversible pulpitis in the two groups and no statistically significant difference was found between types of teeth with irreversible pulpitis [*p*=0.123].

**Figure 1:**
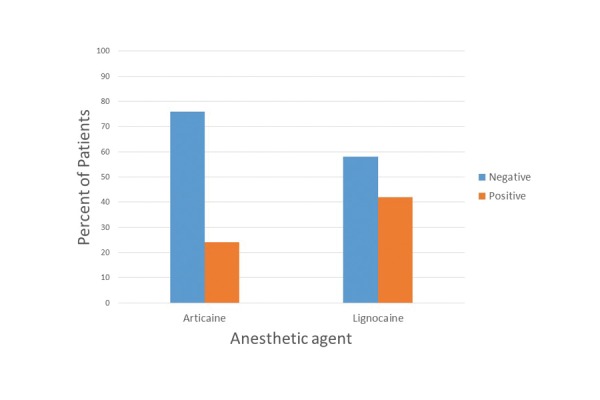
Bar graph of the occurrence of pain (percent) after the respective IAN block solutions.

All 100 patients [100%] reported subjective lip anesthesia 10 minutes after the IAN block. Before the pulpectomy procedure, 38 patients [76%] of the articaine group and 29 patients [58%] of the lidocaine group exhibited pulpal anesthesia (Fig. 1), i.e., a negative response to electrical stimuli generated with an electric pulp tester. However, this slight difference between the 2 experimental groups was not statistically significant [*P*= 0.056].

**Figure 2:**
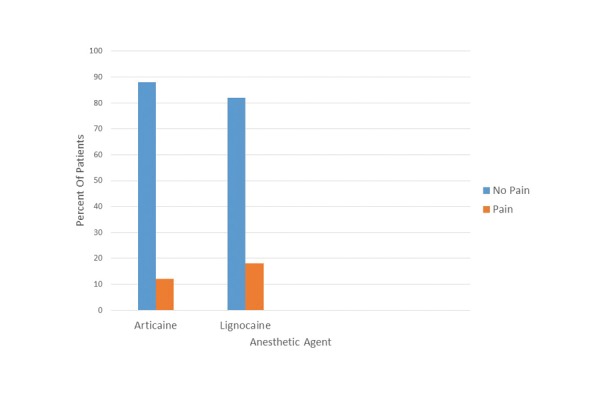
Bar graph of responses to the pulp tester (percent) after the respective IAN block solutions.

During the pulpectomy, 6 patients of the articaine group [12%] and 9 patients of the lidocaine group [18%] reported pain [pain scores 2 and 3]. Again, this difference was not statistically significant [*P* = 0.401] (Fig. 2).

## Discussion

In our study there are no significant differences between the patients of the 2 test groups [articaine versus lidocaine solution] regarding their gender, age, and type of posterior tooth with irreversible pulpitis. Consequently, any potential effects of these parameters can be minimized or even neglected, and the results obtained with both anesthetic solutions can be directly compared.

The Anesthetic solution of 2% lidocaine with 1:80,000 epinephrine was used in the study as these cartridges are most commonly used the institution in which the study was carried. The concentration of epinephrine does not affect the degree of anesthesia obtained with 2% lidocaine for inferior alveolar nerve block (15).

Our study demonstrates that although both local anesthetic solutions always resulted in profound lip anesthesia 10 minutes after their application, pulpal anesthesia is not necessarily achieved. Only 76% of the patients in the articaine group and 58% of the patients in the lidocaine group exhibited pulpal anesthesia, i.e., a negative response to electrical stimuli generated by an electric pulp tester which is in confirmation to the results of another study that had investigated the effect of articaine and lidocaine used for inferior alveolar nerve block (1).

In the articaine group, 12% of the patients experienced pain during the pulpectomy. The percentage of patients reporting pain during the treatment was only insignificantly higher in the lidocaine group [18%].

The results of the study point to the fact that neither of the local anesthetic solutions used guarantees a complete absence of pain during pulpectomy. Although the success rate is slightly more with the articaine solution than with the lidocaine solution, this difference was not statistically significant. Although 42% of all assessed posterior teeth had responded negatively to the electric pulp test, the patients reported pain during the subsequent endodontic treatment. The similarities between the results of this study and those by other authors give rise to the assumption that a negative response of symptomatic teeth with pulp inflammation to the pulp tester is no reliable indicator for analgesia (16,17).

In this study the anesthesia was defined as successful when the dentist accessed the pulp chamber without pain being reported by the patient [pain scores 0 or 1] measured by the scale made for measuring visual analogue scale. In these cases, the pulpectomy procedure was continued. Pain scores of 2 or 3 classified the IAN block as unsuccessful.

So far, only two other studies, performed by Claffey *et al.* 12 and Tortamano *et al.* (13), compared the efficacy of IAN blocks containing 4% articaine with 1:100,000 epinephrine with those containing 2% lidocaine with 1:100,000 epinephrine in patients with irreversible pulpitis. In the study by Claffey *et al.* (12), the success rates were 24% [9 of 37 patients] for IAN blocks with articaine solution and 23% [8 of 35 patients] for lidocaine solution, while in the study by Tortamano *et al.* the success rates were 65% with articaine solutions and 45% with lidocaine solutions. In both the studies the sample size was small and this may have been the reason for the statistically insignificant difference in the two with articaine showing slightly better result than lidocaine.

So, in our study with large sample size of 100 patients was taken, the success rates were 88% with articaine and 82% with lidocaine which are higher than the previous studies. These differences in success rates could be also due to potential population differences between both studies.

Also, the dosage used by Claffey *et al.* was 2.2 ml while that used by Tortamano *et al.* was 3.6 ml. The dose used in our study was 1.8 ml as recommended by C. Richard Bennett (18) and it is the dosage commonly used [1 cartridge] for inferior alveolar nerve blocks in the institution.

The increase in dosage of local anesthetic also increases its efficacy is not entirely supported by the literature. It has been postulated that once the effective volume of local anesthetic is attained, no further benefit can be achieved through a further increase in dosage. In the case of 2% lidocaine with adrenaline, this effective volume has been reported to be upto 2.0 ml of solution (19).

The superiority of articaine over lidocaine for inferior alveolar nerve blocks in patients with irreversible pulpitis once again could not be statistically corroborated by the clinical study even with a large sample size. Both solutions presented a similar behaviour, and both were not entirely efficient in controlling pain during the treatment of irreversible pulpitis. Since this study showed no difference in the efficacy of both the dental anesthetic agents in controlling pain during the treatment of irreversible pulpitis, articaine which has lesser toxicity could be substituted with lidocaine as dental anesthetic agent for IAN blocks in patients with irreversible pulpitis.

More number of such studies is required to make Articaine as popular anesthetic agent for dental treatment procedures.
